# Soot Combustion over Cu–Co Spinel Catalysts: The Intrinsic Effects of Precursors on Catalytic Activity

**DOI:** 10.3390/ijerph192214737

**Published:** 2022-11-09

**Authors:** Chunlin Zhou, Xinbo Zhu, Fei Zhang, Xinbao Li, Geng Chen, Zijian Zhou, Guohua Yang

**Affiliations:** 1 Faculty of Maritime and Transportation, Ningbo University, Ningbo 315211, China; 2 State Key Laboratory of Coal Combustion, School of Energy and Power Engineering, Huazhong University of Science and Technology, Wuhan 430074, China

**Keywords:** soot combustion, spinel catalyst, precursor, transition metal oxides, catalytic activity

## Abstract

In this work, a series of CuCo_2_O_4_-*x* (*x* = N, A and C) catalysts were synthesized using different metal salt precursors by urea hydrothermal method for catalytic soot combustion. The effect of CuCo_2_O_4_-*x* catalysts on soot conversion and CO_2_ selectivity in both loose and tight contact mode was investigated. The CuCo_2_O_4_-N catalyst exhibited outstanding catalytic activity with the characteristic temperatures (T_10_, T_50_ and T_90_) of 451 °C, 520 °C and 558 °C, respectively, while the CO_2_ selectivity reached 98.8% during the reaction. With the addition of NO, the soot combustion was further accelerated over all catalysts. Compared with the loose contact mode, the soot conversion was improved in the tight contact mode. The CuCo_2_O_4_-N catalysts showed better textural properties compared to the CuCo_2_O_4_-A and CuCo_2_O_4_-C, such as higher specific surface areas and pore volumes. The XRD results confirmed that the formation of a CuCo_2_O_4_ crystal phase in all catalysts. However, the CuO crystal phase only presented in CuCo_2_O_4_-N and CuCo_2_O_4_-A. The relative contents of Cu^2+^, Co^3+^ and O_ads_ on the surface of CuCo_2_O_4_-*x* (*x* = N, A and C) catalysts were analyzed by XPS. The CuCo_2_O_4_-N catalyst displayed the highest relative content of Cu^2+^, Co^3+^ and O_ads_. The activity of catalytic soot combustion showed a good correlation with the order of the relative contents of Cu^2+^, Co^3+^ and O_ads_. Additionally, the CuCo_2_O_4_-N catalyst exhibited lower reduction temperature compared to the CuCo_2_O_4_-A and CuCo_2_O_4_-C. The cycle tests clarified that the copper–cobalt spinel catalyst obtained good stability. In addition, based on the Mars–van Krevelen mechanism, the process of catalytic soot combustion was described combined with the electron transfer process and the role of oxygen species over CuCo_2_O_4_ spinel catalysts.

## 1. Introduction

Soot particles are emitted from incomplete combustion of hydrocarbon fuels in diesel engines [1]. Mitigating soot particle emissions from diesel engines has raised much attention due to the negative impact of soot particles on human health and environmental protection [2,3]. A diesel particulate filter (DPF) is the most effective after-treatment technology for soot particles from diesel engines. However, a common issue of pore blockage over DPFs after a period of use should be addressed [4]. One of the most effective measures to improve soot oxidation is to coat the DPF with catalysts and form a catalyzed DPF (CDPF) [5]. CDPFs with highly active catalysts can accelerate soot oxidation and lower the ignition temperatures.

Various catalysts have been proposed and optimized to accelerate the catalytic soot combustion, while Pt-based catalysts have shown superb soot oxidation performance under practical conditions [6,7]. However, due to the high cost of noble metals, transition metal-based catalysts have been intensively studied for catalytic soot oxidation more recently [8]. Copper–cobalt catalysts play an important role as substitutes for noble metals in the field of catalytic soot combustion due to their outstanding redox properties and thermal stability [9]. Zhang et al. reported that a spinel-type CuCo_2_O_4_ catalyst showed a lower T_50_ temperature (574 °C) of catalytic soot combustion than that of Co_3_O_4_ catalysts (580 °C) due to its higher relative content of Co^3+^ species [10]. Jampaiah et al. found that a CuCo-MnO_2_ catalyst exhibited a lower T_90_ temperature (485 °C) of catalytic soot combustion than that of NiCo-MnO_2_ catalysts (513 °C) under the loose contact mode due to more oxygen vacancies and active sites on the catalyst surfaces [11]. Zhang et al. tested the stability of CuCo_2_O_4_ spinel catalysts in four consecutive soot combustion cycles and found that the values of T_50_ for catalytic soot combustion increased from 539 °C to 544 °C, 547 °C and 549 °C, respectively [12].

Recently, the effect of metal salt precursor on the textural and redox properties of catalysts was found to play a key role in catalytic activity and product selectivity [13]. Yang et al. found that the catalytic activity of selective acetylene hydrogenation at 50 °C over the Pd/Al_2_O_3_ catalyst using acetate as the Pd precursor reached 78.6%, which was 31.1% higher than that using chloride precursors since Cl residuals existed in the form of ${PdCl}_{4}^{2-}$, resulting in the decrease of the electron density of Pd atoms [14]. Wang et al. reported that the n-hexadecane conversion over Pt/ZSM-22 catalysts with Pt(NH_3_)_4_Cl_2_ precursors was ~9.1% higher compared to that over catalysts using (Pt(NO_3_)_2_ and H_2_PtCl_6_ as the precursors due to the higher platinum dispersion [15]. Yun et al. found that the Ni/Al_2_O_3_ catalyst using acetate salt precursors achieved the highest activity (85.6%) in the steam reforming of acetic acid at 450 °C, while the catalytic activity of the catalyst prepared from chloride salts was only 38.7% [16]. These results indicated that the role of precursors should be preferentially considered during the designing and preparation of heterogeneous catalysts.

In this work, copper–cobalt spinel oxides were prepared with a urea hydrothermal method using different copper and cobalt precursors (nitrate, chloride and acetate). The physicochemical properties of these catalysts were characterized by N_2_ adsorption–desorption, X-ray diffraction (XRD), scanning electron microscopy (SEM), X-ray photoelectron spectroscopy (XPS), temperature-programed reduction of H_2_ (H_2_-TPR) and temperature-programed desorption of O_2_ (O_2_-TPD). The catalytic activity of soot combustion was investigated by temperature-programed oxidation (TPO) experiments under various reaction conditions.

## 2. Experimental Study

### 2.1. Catalyst Preparation

In this work, a series of CuCo_2_O_4_-*x* catalysts was synthesized by urea hydrothermal methods with different precursors, where *x* represented the types of copper and cobalt precursors, e.g., the nitrates (N), acetate (A) and chloride (C), respectively. All chemicals used were of analytical reagent grade and purchased from Macklin Co., Ltd. (Shanghai, China). Taking CuCo_2_O_4_-N as an example, 0.02 mol Cu(NO_3_)_2_·3H_2_O, 0.04 mol Co(NO_3_)_3_·6H_2_O and 0.06 mol CH_4_N_2_O were dissolved in 100 mL of deionized water and stirred for 1 h at room temperature. Afterwards, the mixed solution was transferred to a polytetrafluoroethylene-lined high pressure reactor placed in a thermostat at 150 °C for hydrothermal reaction. After 12 h, the resulting solution was filtered to obtain a precipitate. The precipitate was then washed three times with deionized water and dried in an oven at 110 °C overnight. Finally, the precipitate was calcined in a muffle furnace at 700 °C for 6 h and sieved to 40–60 meshes. The prepared catalysts were denoted as CuCo_2_O_4_-N, CuCo_2_O_4_-A and CuCo_2_O_4_-C, respectively.

### 2.2. Catalyst Characterizations

N_2_ adsorption–desorption analysis was conducted at 77 K to determine the specific surface area, pore size distribution and average pore diameter of the catalysts using TriStar II 3020 (Micromeritics, Norcross, GA, USA). The specific surface areas were calculated using the Brunauer–Emmett–Teller (BET) method. Total pore volume and pore size distribution were calculated via the Barrett–Joyne–Halenda (BJH) method at the relative pressure of *p*/*p*_0_ = 0.99. The crystal structure of catalysts was observed by X-ray diffraction (XRD) using a diffractometer system (D-Max 2000, Rigaku, Tokyo, Japan) with Cu-Kα radiation operating at 40 kV and 30 mA. All catalysts were scanned in the range of 10° to 80° with a step size of 0.02°. A scanning electron microscopy (SEM) instrument (JSM-7001F, JEOL, Tokyo, Japan) was used to observe the surface morphology of catalysts at an accelerating voltage of 10 kV. The X-ray photoelectron spectra (XPS) were measured with Thermo Escalab 250Xi equipment with monochromatic Al-Ka X-ray radiation at 150 W. All binding energies were calibrated using the C 1s photoelectron peak at 284.8 eV. The redox properties of all catalysts were analyzed using the temperature-programed reduction of the H_2_ (H_2_-TPR) apparatus (Autochem II 2920, Micromeritics, Norcorss, GA, USA). To remove impurities, 16 mg catalysts were pretreated at 250 °C for 1 h and cooled down to room temperature before each test. Then, the catalysts were heated from room temperature to 800 °C at a heating rate of 10 °C·min^−1^ in a 30 mL·min^−1^ feeding gas flow (10 vol.% H_2_/Ar). The amount of H_2_ consumption was calculated based on the H_2_-TPR profiles. The profiles of the temperature-programed desorption of O_2_ (O_2_-TPD) were performed using the same apparatus as that for H_2_-TPR. Before the measurement, 200 mg of catalysts were pretreated in an He stream at 200 °C for 1 h and cooled down to room temperature. Then, the adsorption of O_2_ was conducted at 70 °C for 1 h in a gas mixture of 3 vol.% O_2_/He (30 mL·min^−1^). Subsequently, the sample was purged by a flowing pure He stream to remove excessive and weakly adsorbed O_2_. Finally, the sample was heated to 800 °C with a heating rate of 10 °C·min^−1^ in a pure He flow (30 mL·min^−1^), and the desorption profile was recorded.

### 2.3. Experimental System

**Figure 1:**
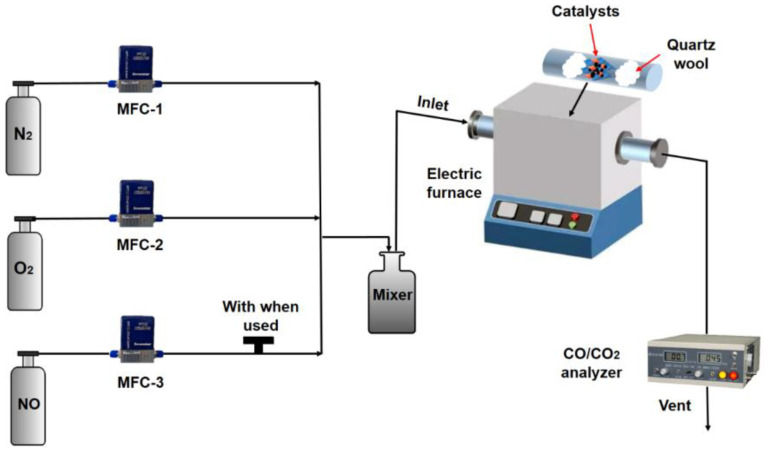
Schematic diagram of the experimental setup.

Figure 1 shows a schematic diagram of the experimental setup. The catalytic activity of the CuCo_2_O_4_-*x* (*x* = N, C and A) catalysts was evaluated by temperature-programed oxidation (TPO) experiments. Printex-U (Degussa, with the size of 20–30 nm) was used as the model soot in this study. For each test, 180 mg of catalyst powder was mixed with 20 mg of soot in loose (mixing with a spatula for 5 min) or tight (grinding in an agate mortar for 5 min) contact mode. Then, the resulting soot–catalyst mixtures (200 mg) were mixed with 400 mg of inert silica (40–60 meshes) for another 5 min to avoid the formation of hot spots during the reaction. The experimental procedure was as follows: Firstly, the mixture of catalyst and inert silica after reaction was collected from the quartz tube and then placed in an agate mortar. Secondly, 20 mg of soot was mixed with the obtained mixture of the catalyst and inert silica after reaction in loose contact mode. Finally, the above obtained mixture of soot, catalyst and inert silica was filled back into the quartz tube for next catalytic soot combustion reaction cycle. The reaction temperature was increased from 50 °C to 700 °C at a heating rate of 5 °C·min^−1^.

All gas streams from the gas cylinders were regulated by mass flow controllers (Sevenstars D07-B, Beijing, China). The mixed gases (10 vol.% O_2_ with balanced N_2_) were fed into the reactor at the flow rate of 200 mL·min^−1^ during the TPO experiment. In addition, 1000 ppm NO was added into the feeding gas to investigate the effect of NO on catalytic soot combustion when necessary. The outlet concentrations of CO and CO_2_ were monitored online using an infrared (IR) gas analyzer (GXH-3010/3011AE, Huayun, Beijing, China) with the accuracy of ±3%. The temperatures at which 10%, 50% and 90% of the soot was oxidized (denoted as T_10_, T_50_ and T_90_, respectively) were recorded as indicators of the catalytic activity. Soot conversion (denoted as α) and CO_2_ selectivity (denoted as SCO_2_) were calculated by integrating the CO and CO_2_ concentration curves with time as follows:

(1) $$\alpha \;(\%)=\frac{\int_{0}^{t}({\left[CO_{2}\right]}_{out}+{\left[CO\right]}_{out})dt}{M}\times 100\%$$

(2) $$S_{CO_{2}}(\%)=\frac{\int_{0}^{t}{\left[CO\right]}_{out}dt}{\int_{0}^{t}({\left[CO_{2}\right]}_{out}+{\left[CO\right]}_{out})dt}\times 100\%$$

where [CO_2_]_out_ and [CO]_out_ are the real-time concentrations of CO and CO_2_ at the reactor outlet, respectively, and M is the weight of the initially packed soot.

## 3. Results and Discussion

### 3.1. Textural Properties of the Catalysts

The specific surface area (S_BET_), pore volume and pore size of CuCo_2_O_4_-*x* (*x* = N, A and C) catalysts are obtained with a N_2_ adsorption–desorption experiment (Table 1). The CuCo_2_O_4_-N catalyst shows the largest specific surface area of 2.1 m^2^·g^−1^, followed by CuCo_2_O_4_-A (2.0 m^2^·g^−1^) and CuCo_2_O_4_-C (1.2 m^2^·g^−1^). The pore volumes of CuCo_2_O_4_-N, CuCo_2_O_4_-A and CuCo_2_O_4_-C catalysts are 4.5 mm^3^·g^−1^, 4.3 mm^3^·g^−1^ and 2.5 mm^3^·g^−1^, respectively. The specific surface area and pore volume of the CuCo_2_O_4_ catalysts prepared with nitrate and acetate metal salt show no significant differences, while the specific surface area and pore volume of CuCo_2_O_4_-C catalyst dramatically decreases. Yu et al. also reported the formation of hydrochloric acid from chloride salts during calcination, which may inhibit the formation of developed pore systems and negatively affect the specific surface area [16]. Compared with conventional metal oxides and supported catalysts, the specific surface area, pore volume and average pore size of the prepared Cu–Co spinel catalysts are rather low. The results could be ascribed to the formation of the well-crystallized spinel structure under high calcination temperature (700 °C) [12,17].

**Figure 2:**
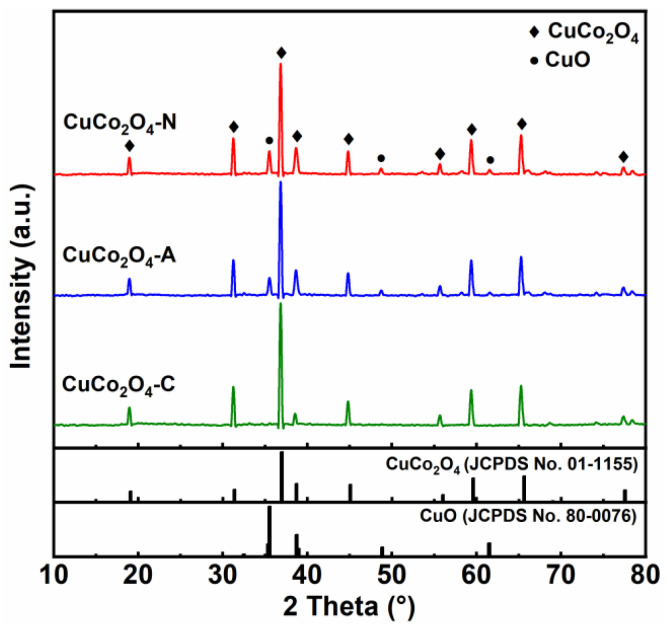
XRD patterns of the CuCo_2_O_4_-*x* (*x* = N, A and C) catalysts.

Figure 2 shows the XRD patterns of the CuCo_2_O_4_-*x* (*x* = N, A and C) catalysts. Sharp and intense diffraction peaks of copper–cobalt spinel phases are observed for all samples, suggesting the formation of well-crystallized structures at high calcination temperature of 700 °C. The diffraction peaks observed at 19.1°, 31.4°, 36.9°, 45.1°, 56.0°, 59.6°, 68.9° and 77.5° are attributed to tetragonal spinel crystalline of CuCo_2_O_4_ (JCPDS No. 01-1155) [18]. Meanwhile, metal oxide CuO phase (JCPDS No. 80-0076) is observed at 35.6°, 48.6° and 61.7° over CuCo_2_O_4_-N and CuCo_2_O_4_-A catalysts [19]. No distinct CuO phase is found on the CuCo_2_O_4_-C catalyst. Based on the characteristic peak of CuCo_2_O_4_ (3 1 1) crystal face, the crystal size of the CuCo_2_O_4_-*x* catalysts were calculated using the Scherrer equation (Table 1). The crystal size of the CuCo_2_O_4_-N catalyst (38.0 nm) is slightly smaller than that of CuCo_2_O_4_-A and CuCo_2_O_4_-C catalysts. Wen et al. also found that the Cl^-^ coordination anion was an important contributor to the formation of larger clusters of CuCo_2_O_4_ [20].

**Figure 3:**
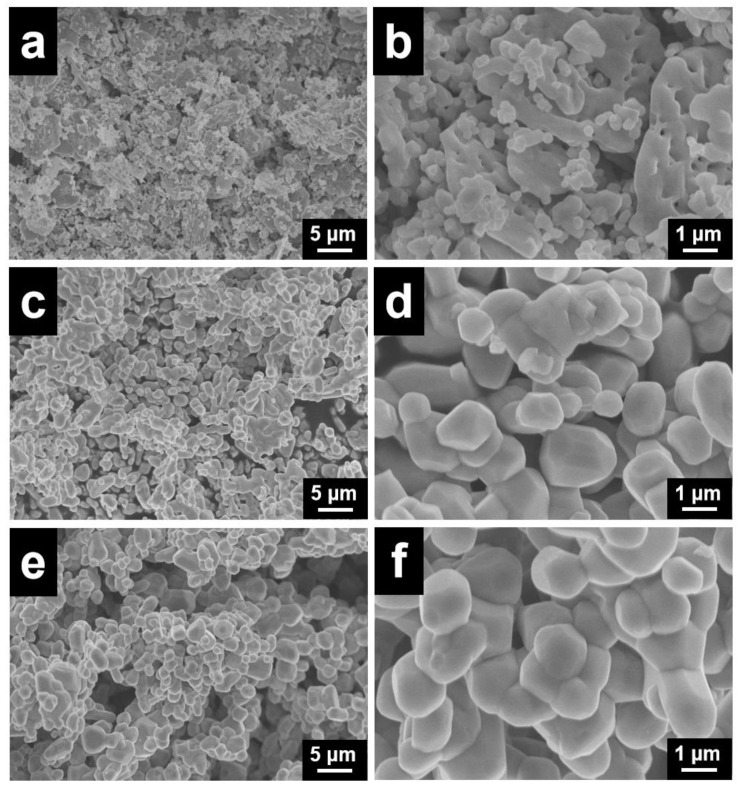
SEM images of the CuCo_2_O_4_-N (**a**,**b**), CuCo_2_O_4_-A (**c**,**d**) and CuCo_2_O_4_-C (**e**,**f**) catalysts.

Figure 3 shows the representative SEM images of the CuCo_2_O_4_-*x* (*x* = N, A and C) catalysts. The CuCo_2_O_4_-N catalyst exhibits a sheetlike morphology with tiny spherical particles on its surface. The stacking of spherical particles and its sheetlike morphologies promote the formation of porous structures (Figure 3a). The phenomenon of particle agglomeration is exhibited over the CuCo_2_O_4_-A and CuCo_2_O_4_-C catalyst (Figure 3c,e). As shown in Figure 3b, the spherical particles are dispersed on the sheetlike morphology and formed a dendritic structure. However, tiny spherical particles are hardly generated on the surfaces of the CuCo_2_O_4_-A and CuCo_2_O_4_-C catalysts (Figure 3d,f), while the agglomeration of bulk particles might have resulted in the blockage of the pore systems. These tiny spherical particles can improve the contact between the catalyst and soot particles, which can facilitate the utilization of the catalyst active sites for catalytic soot combustion. The particle sizes of CuCo_2_O_4_-A and CuCo_2_O_4_-C catalysts increased obviously compared to the CuCo_2_O_4_-N catalysts. 

### 3.2. Redox Properties of the Catalysts

**Figure 4:**
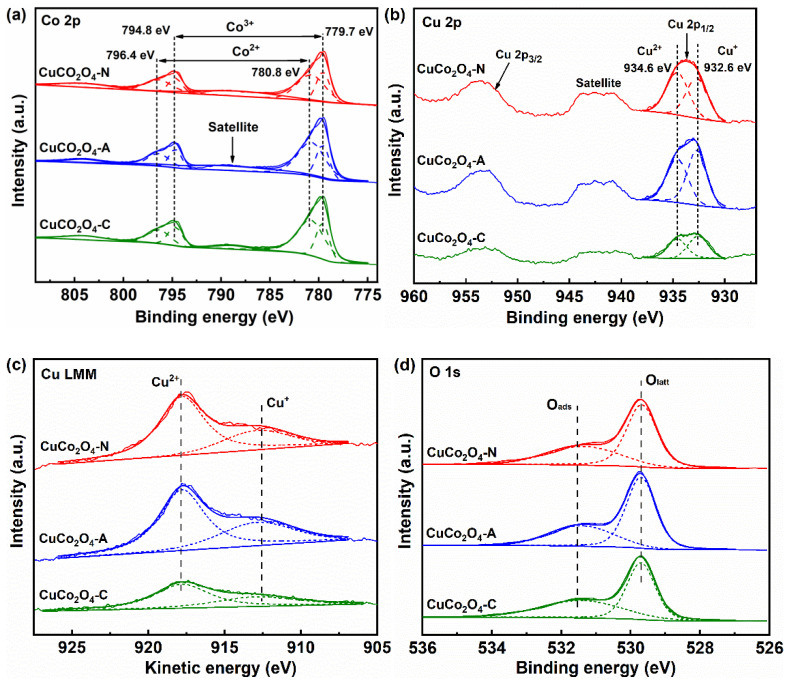
XPS spectra of the CuCo_2_O_4_-*x* (*x* = N, A and C) catalysts: (**a**) Co 2p; (**b**) Cu 2p; (**c**) Cu LMM; (**d**) O 1s.

The Co 2p spectra of the CuCo_2_O_4_-*x* (*x* = N, A and C) catalysts are shown in Figure 4a. The peaks of Co 2p_3/2_ and Co 2p_1/2_ are observed in the range of 776.0–784.0 eV and 792.0–800.0 eV, respectively [21]. The peaks between 784.0–792.0 eV and 800.0–808.0 eV are attributed to the satellite peaks of Co^2+^ [22,23]. The Co^3+^ and Co^2+^ signals are obtained after the deconvolution of the Co 2p_3/2_ and Co 2p_1/2_ spectra. The peaks centered at 779.7 eV and 794.8 eV correspond to the Co^3+^ species, while the peaks located at 780.8 eV and 796.4 eV belong to the Co^2+^ species [24]. The Cu 2p spectra of all catalysts are also given in Figure 4b. The peaks observed at 932.6 eV belong to the reduced Cu species (Cu^+^ or Cu^0^), while the prominent signals at 934.6 eV are ascribed to the Cu^2+^ species [25]. Additionally, the satellite peaks between 937.0 eV to 946.0 eV also confirm the existence of divalent Cu species [19]. Figure 4c shows the Cu LMM Auger spectra of all CuCo_2_O_4_ catalysts. The peaks at the kinetic energy of 912.6 eV correspond to the Cu^+^ species, while the peaks at 917.8 eV are attributed to the Cu^2+^ species [26]. However, Cu^0^ species are not observed on the Cu LMM Auger spectrum. These results suggest that the reduced copper species on the surfaces of CuCo_2_O_4_-*x* (*x* = N, A and C) catalysts mainly exists as Cu^+^. The de-convoluted XPS signals of O 1s are shown in Figure 4d. Two types of oxygen species present on the surface of the CuCo_2_O_4_ catalysts. The peaks around 529.8 eV correspond to the lattice oxygen species (O_latt_), while the peaks around 531.4 eV are attributed to the adsorbed oxygen species (O_ads_) [27].

The relative contents of Cu^2+^ and Co^3+^ on the surface of CuCo_2_O_4_-*x* catalysts are given in Table 2. The highest relative content of Co^3+^/Co_total_ (38.4%) is obtained over the CuCo_2_O_4_-N catalyst, followed by CuCo_2_O_4_-A (35.1%) and CuCo_2_O_4_-C (33.8%). Moreover, the highest relative content of Cu^2+^/Cu_total_ is also achieved over the CuCo_2_O_4_-N catalyst (51.7%), followed by CuCo_2_O_4_-A (48.2%) and CuCo_2_O_4_-C (47.8%). The highest relative content of O_ads_/(O_ads_ + O_latt_) (46.8%) is found over the CuCo_2_O_4_-N catalyst, followed by CuCo_2_O_4_-A (37.2%) and CuCo_2_O_4_-C (36.5%) (Table 2). The adsorbed oxygen species were more chemically active than lattice oxygen in catalytic soot combustion, indicating a better soot conversion performance over the CuCo_2_O_4_-N catalyst with more O_ads_ species [28].

**Figure 5:**
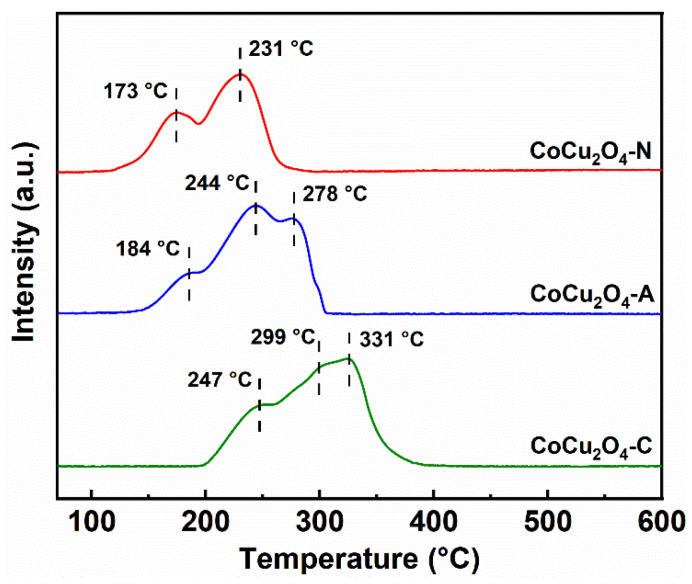
H_2_-TPR profiles of the CuCo_2_O_4_-*x* (*x* = N, A and C) catalysts.

For the CuCo_2_O_4_-N catalyst, two reduction peaks are observed (Figure 5). The first reduction peak in the range of 150–200 °C is attributed to the reduction of aggregated CuO, while the second peak between 200 °C and 250 °C can be ascribed to the reduction of Cu–Co mixed oxides [29]. The CuCo_2_O_4_-A catalyst exhibits shoulder peaks at 200–300 °C, the first reduction peak (244 °C) corresponds to the reduction of Cu–Co mixed oxides, and the latter reduction peak at 278 °C represents the reduction of Co^3+^ to Co^2+^ [30]. The CuCo_2_O_4_-C catalyst also shows two major peaks between 250 °C and 350 °C. The weak reduction peak at 299 °C is ascribed to the reduction of Co^3+^ to Co^2+^, while the reduction peak at 331 °C represents the reduction of Co^2+^ to Co^0+^ [31]. These results suggest that CuCo_2_O_4_-N catalyst has better reducibility at relatively low temperatures. The amount of H_2_ consumption was in the order of CuCo_2_O_4_-C > CuCo_2_O_4_-A > CuCo_2_O_4_-N. Although the CuCo_2_O_4_-C catalysts show the higher H_2_ consumption (14.5 mmol·g^−1^), the H_2_ consumption of the CuCo_2_O_4_-N catalyst is likely mainly concentrated in the low temperature range (150–250 °C). Therefore, the distribution of oxygen species was further investigated.

**Figure 6:**
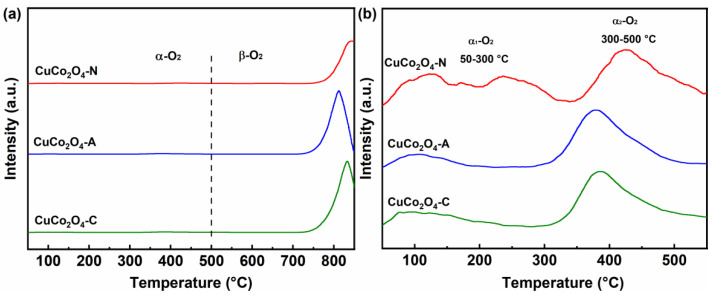
O_2_-TPD profiles (**a**) and the enlarged O_2_-TPD profiles in the temperature range of 50 °C to 550 °C (**b**) of the CuCo_2_O_4_-*x* (*x* = N, A and C) catalysts.

Figure 6a shows the O_2_-TPD profiles of the CuCo_2_O_4_-*x* (*x* = N, A and C) catalysts. The oxygen desorption peaks below 500 °C belong to adsorbed oxygen species (e.g., O_2_, $O_{2}^{-}$ and O^-^, labeled as α-O_2_) [32], while the oxygen desorption peaks between 600 °C and 850 °C belong to the lattice oxygen species (O^2−^, labeled as β-O_2_) [33]. Figure 6b shows the enlarged O_2_-TPD profiles in the temperature range of 50 °C to 550 °C. The oxygen desorption peaks within 50–300 °C and 300–500 °C correspond to the physically adsorbed oxygen (α_1_-O_2_) and chemically adsorbed oxygen (α_2_-O_2_) species, respectively [34,35]. Generally, the adsorption of oxygen followed the procedure of O_2_→ $O_{2}^{-}$→O^-^→O^2−^ [27]. The CuCo_2_O_4_-N catalyst shows an extra oxygen desorption peak in the temperature range of 200 °C to 250 °C compared to the CuCo_2_O_4_-A and CuCo_2_O_4_-C. The extra oxygen desorption peak could be attributed to physically adsorbed oxygen (α_1_-O_2_), indicating that the catalysts had better oxygen mobility [36]. Similarly, Li et al. also reported that the CuCo_2_O_4_ and NiCo_2_O_4_ catalysts showed an extra oxygen desorption peak in the temperature range of 200 °C to 250 °C compared to the ZnCo_2_O_4_. The catalytic performance of CuCo_2_O_4_ and NiCo_2_O_4_ catalysts were found to be far superior to that of ZnCo_2_O_4_ in toluene combustion [7]. The desorption amount of α_1_-O_2_ and α_2_-O_2_ species were calculated according to the desorption peaks in the O_2_-TPD profiles. As shown in Table 2, the amount of O_2_ desorption was in the order of CuCo_2_O_4_-N (41.2 μmol·g^−1^) > CuCo_2_O_4_-A (27.1 μmol·g^−1^) > CuCo_2_O_4_-C (25.6 μmol·g^−1^), which confirms the existence of more adsorbed oxygen species over the CuCo_2_O_4_-N catalyst.

### 3.3. Activity of CuCo_2_O_4_-x for Soot Conversion

#### 3.3.1. Soot Conversion in O_2_/N_2_

**Figure 7:**
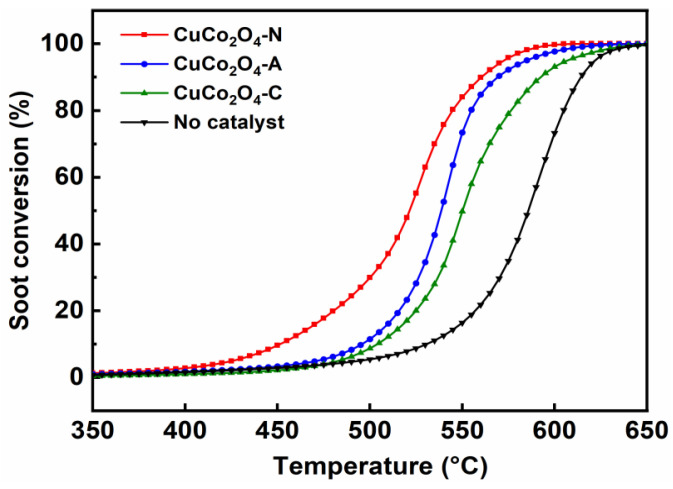
Soot conversion over the CuCo_2_O_4_-*x* (*x* = N, A and C) catalysts under loose contact mode under the carrier gas of 10 vol.% O_2_ with balanced N_2_.

The catalytic activity of CuCo_2_O_4_-*x* (*x* = N, A and C) catalysts for soot combustion were studied using a TPO experiment under the loose contact mode. Firstly, the soot conversion was investigated under the carrier gases of 10 vol.% O_2_ balanced with N_2_. The characteristic temperatures (T_10_, T_50_ and T_90_) of soot conversion are 530 °C, 586 °C and 614 °C, respectively in the absence of a catalyst, which is much higher than those in the presence of the CuCo_2_O_4_ catalysts (Figure 7 and Table 3). The catalysts prepared with different metal salt precursors exhibit different catalytic activity in the soot conversion. Among them, the CuCo_2_O_4_-N catalyst achieves the highest soot conversion, while the values of T_10_, T_50_ and T_90_ are 451 °C, 520 °C and 558 °C, respectively. The T_50_ values for the CuCo_2_O_4_-*x* (*x* = N, A and C) catalysts followed the order of CuCo_2_O_4_-N (520 °C) < CuCo_2_O_4_-A (539 °C) < CuCo_2_O_4_-C (550 °C) < no catalysts (586 °C). The CO_2_ selectivity is also improved remarkably from 81.8% over the CuCo_2_O_4_-C to almost 100% over the CuCo_2_O_4_-N catalysts.

The CuCo_2_O_4_-N and CuCo_2_O_4_-A catalysts exhibit larger specific surface area and pore volume compared to the CuCo_2_O_4_-C catalyst. It is widely recognized that a larger specific surface area could facilitate the contact between gaseous reactants (e.g., O_2_, NO and NO_2_) and the catalyst [37,38]. SEM images further confirmed the generation of more developed pore structure systems of the CuCo_2_O_4_-N catalyst compared to the CuCo_2_O_4_-A and CuCo_2_O_4_-C catalysts. Fang et al. reported that a perovskite-type macro/mesoporous La_1–*x*_K*_x_*FeO_3–δ_ catalysts with large specific surface area and pore volume could improve the utilization of catalytic sites in the soot combustion reaction [39]. Furthermore, the presence of an appropriate amount of single metal oxide CuO on the CuCo_2_O_4_ surface could contribute to the crystal lattice distortion of the spinel phase and promote the formation of a defect structure, thereby improving the catalytic activity of the soot combustion reaction [40].

The redox properties of CuCo_2_O_4_-*x* catalysts played a crucial role in catalytic soot combustion. The higher relative content of Co^3+^/Co_total_ (38.4%) and Cu^2+^/Cu_total_ (51.7%) obtained over CuCo_2_O_4_-N catalysts were much higher compared to the CuCo_2_O_4_-A (35.1% and 48.2%) and CuCo_2_O_4_-C (33.8% and 47.8%), respectively. Spinel-type CuCo_2_O_4_ catalysts possessed outstanding redox properties since the synergistic effects between Co^3+^ and Cu^2+^ species could enhance the adsorption-activation properties of oxygen species for soot conversion [41]. Abundant Co^3+^ species would increase the anionic defects on catalyst surfaces, leading to the formation of more oxygen vacancies [42]. Zhang et al. reported that the presence of Cu^2+^ species induced structural defects on cobalt oxide and weakened the Co-O bonds, which could facilitate the activation of oxygen and improve the reducibility of CuCo_2_O_4_ [43]. In addition, the CuCo_2_O_4_-N catalysts showed the highest relative content of O_ads_/(O_ads_ + O_latt_) (46.8%) compared to the CuCo_2_O_4_-A (37.2%) and CuCo_2_O_4_-C (36.5%). The O_ads_ species possessed better mobility than O_latt_ species and could participate in catalytic soot combustion via the contact points between catalyst pellets and soot particulates [35]. 

The lower reduction peaks temperature proved that the CuCo_2_O_4_-N catalysts (173 °C) has excellent low temperature reduction performances compared to the CuCo_2_O_4_-A (184 °C) and CuCo_2_O_4_-C (247 °C). The total H_2_ consumption amount of the CuCo_2_O_4_-N catalysts (13.4 mmol·g^−1^) was slightly lower than that of CuCo_2_O_4_-A (13.6 mmol·g^−1^) and CuCo_2_O_4_-C (14.5 mmol·g^−1^). The CuCo_2_O_4_-N catalyst possessed more adsorbed oxygen species (41.2 μmol·g^−1^) compared to the CuCo_2_O_4_-A (27.1 μmol·g^−1^) and CuCo_2_O_4_-C (25.6 μmol·g^−1^). It was suggested that the CuCo_2_O_4_-N catalysts could release more adsorbed oxygen species below 500 °C [44]. Compared with lattice oxygen species, adsorbed oxygen species were more important in soot conversion due to their better oxygen mobility [45]. He et al. also reported that the adsorbed oxygen species released at low temperatures were more important than lattice oxygen species since the lattice oxygen species could be activated and released only at high temperatures [35]. Lower reduction temperature and abundant adsorbed oxygen species may be crucial factors for soot conversion over the CuCo_2_O_4_-N catalyst at lower temperature.

#### 3.3.2. Soot Conversion in NO/O_2_/N_2_

**Figure 8:**
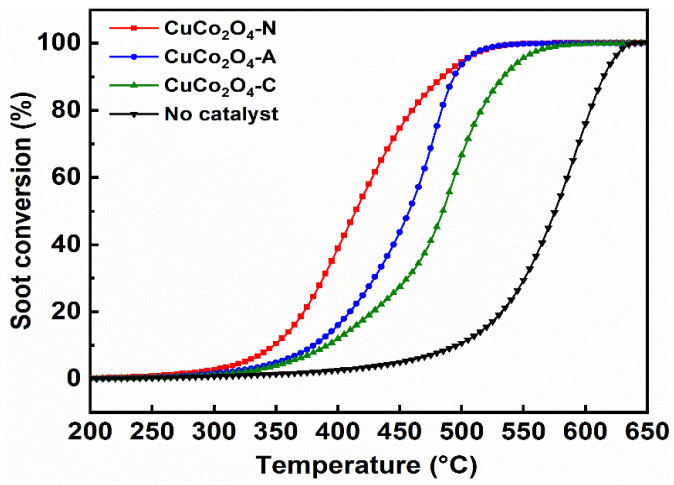
Soot conversion of the CuCo_2_O_4_-*x* (*x* = N, A and C) catalysts under the loose contact mode with 1000 ppm NO.

NO is one of the main components in diesel engine exhaust and has a great effect on soot conversion. Therefore, soot conversion was investigated over the CuCo_2_O_4_-*x* (*x* = N, A and C) catalysts in the presence of 1000 ppm NO (Figure 8 and Table 4). The addition of NO resulted in lower T_10_, T_50_ and T_90_ values regardless of the presence of a catalyst. The T_50_ values for the CuCo_2_O_4_-*x* (*x* = N, A and C) catalysts also follow the order of CuCo_2_O_4_-N (414 °C) < CuCo_2_O_4_-A (458 °C) < CuCo_2_O_4_-C (485 °C) < no catalysts (575 °C). 

Using CuCo_2_O_4_-N as an example, the T_50_ value decreases from 520 °C to 414 °C with NO addition, while the CO_2_ selectivity also decreases from 98.8% to 96.6%. NO could be oxidized with O_2_ to form NO_2_ in gas phase (Reaction (3)) [46]. NO could be also adsorbed on the surface of CuCo_2_O_4_-N catalyst and then oxidized by the active oxygen to form NO_2_ species, while could promote the formation of surface oxygen vacancies (Reaction (4)). NO_2_ is a stronger oxidant and participate in catalytic soot combustion as a mobile gaseous oxidant [46]. Possible pathways for the reaction of NO_2_ with soot are shown in Reaction (5) [47]. NO_2_ could attack the soot on the surface, resulting in the generation of CO and NO, while CO was eventually oxidized by O_2_ to CO_2_, with NO again participating in the NO*_x_*-assisted catalytic soot combustion [37]. However, the slight decrease in CO_2_ selectivity could be attributed to the rapid oxidation of soot by NO_2_, resulting in the incomplete oxidation of soot to CO [48].

(3) $$2NO+O_{2}\to 2NO_{2}$$

(4) $$NO+O^{*}\to NO_{2}+O_{v}$$

(5) $$NO_{2}+C(S\mathrm{oo}t)\to CO+NO$$

where O* represents the active oxygen species, O_v_ represents the surface oxygen vacancy and C(Soot) represents the soot particulate.

#### 3.3.3. Effect of the Contact Mode

**Figure 9:**
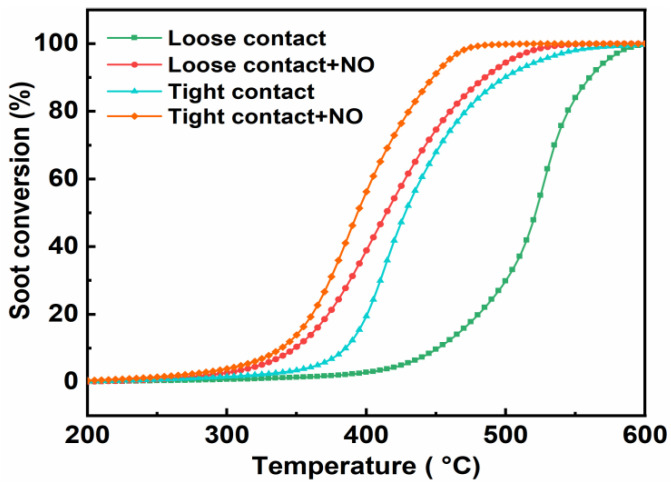
Soot conversion over the CuCo_2_O_4_-N catalyst under the loose and tight contact modes.

The contact mode between the catalyst and soot could greatly affect the activity of soot conversion [41]. The soot conversion and CO_2_ selectivity were investigated under both loose and tight contact modes over the CuCo_2_O_4_-N catalyst (Figure 9 and Table 5). Under the tight contact mode, soot conversion is accelerated at lower temperatures regardless of the presence of NO compared to the loose contact mode, while the CO_2_ selectivity reaches up to 100%. The increase of catalyst–soot contact points in the tight contact mode could contribute to better utilization of active sites on the catalyst surface compared to the loose contact mode [4,27]. Machida et al. found that the utilization/transfer of lattice oxygen species was more efficient under the tight contact mode. Besides, the case of “O_2_ slip” may be decreased in the tight contact mode, which increases the utilization of the released oxygen species to the soot combustion [49]. 

#### 3.3.4. Stability Test

**Figure 10:**
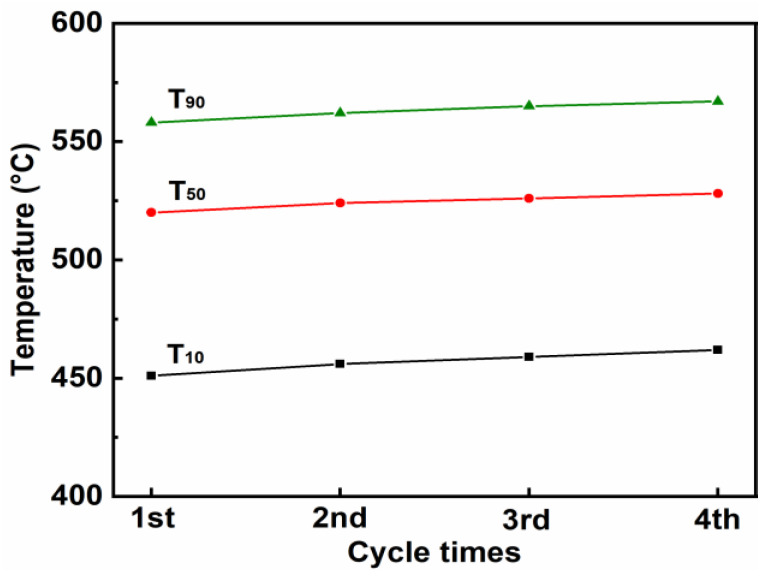
Stability of CuCo_2_O_4_-N catalyst in cycle tests for soot combustion.

**Figure 11:**
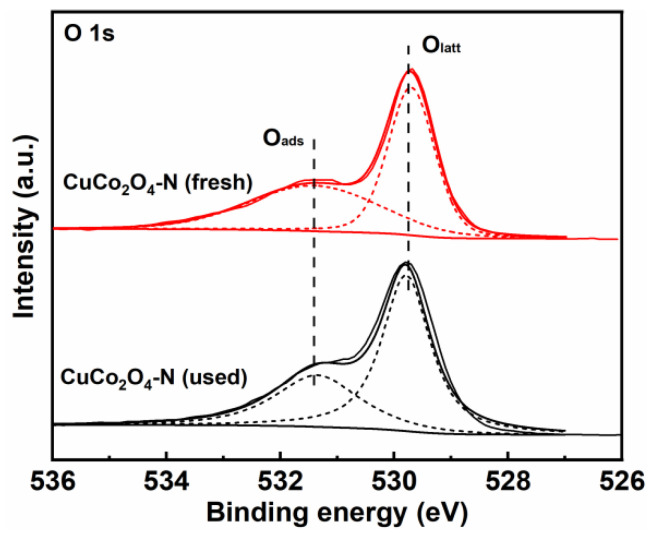
XPS spectra of O 1 s of CuCo_2_O_4_-N catalyst before and after reaction.

The stability test of CuCo_2_O_4_-N catalysts was conducted for four consecutive cycles of soot combustion. As shown in Figure 10, the characteristic temperatures (T_10_, T_50_ and T_90_) increased slightly with the increase in the number of cycles of soot combustion. For examples, the values of T_50_ for catalytic soot combustion for each cycle are 520 °C, 524 °C, 526 °C and 528 °C, respectively. Figure 11 shows the deconvoluted XPS spectra of O 1s for the CuCo_2_O_4_-N catalyst after four consecutive cycles of soot combustion. The relative content of O_ads_/(O_ads_ + O_latt_) of CuCo_2_O_4_-N catalyst decreases from 46.8% to 40.6% (Table 6). The slight decrease in soot catalytic activity may be due to the minor attenuation of the relative content of O_ads_/(O_ads_ + O_latt_). Chen et al. also reported the catalytic soot performance of Ag/Co_3_O_4_ and found that the decrease in adsorbed oxygen species was an important reason for the decrease of catalytic soot combustion [50].

## 4. Reaction Mechanisms for Catalytic Soot Combustion

The potential reaction pathways of catalytic soot combustion over CuCo_2_O_4_ were discussed. The excellent catalytic soot combustion of the copper–cobalt spinel catalyst performance depended on the redox properties of the catalysts [41]. The interactions between Cu and Co species in the CuCo_2_O_4_ catalyst played a crucial role in the redox reactions. Previous studies showed that the redox pairs of Cu^2+^/Cu^+^ and Co^3+^/Co^2+^ were involved in the electron transfer process from Co^3+^ to Cu^2+^ within the Cu^2+^–O–Co^3+^ connections in the copper–cobalt spinel catalysts [31,51]. The Cu^2+^–O–Co^3+^ connections could bridge the oxygen transfer within the structure and reduce the redox potential of the Cu species, which ensures the improvement of reducibility for both Cu and Co oxides in the Cu–Co catalysts (Reaction (6)), facilitating the NO*_x_*-assisted mechanism in the soot combustion reaction [46].

(6) $$Cu^{2+}/Cu^{+}\overset{2e^{-}}{\underset{O^{2-}/O^{-}}{⇄}}Co^{3+}/Co^{2+}$$

Catalytic soot combustion over the copper–cobalt spinel catalyst followed the Mars–van Krevelen (MvK) mechanism, in which the abundance of active oxygen species directly determined the performance of catalytic soot combustion [5]. At the beginning of soot conversion, plenty of active oxygen species on the catalyst surface would come into contact with the soot, resulting in soot combustion at a relatively low temperature and the subsequent formation of oxygen vacancies (O_v_) (Reaction (7)) [49]. As the reaction proceeded, the consumed reactive oxygen species could be replenished in two ways. Firstly, the reduction of high-valence Cu^2+^ and Co^3+^ to low-valence Cu^+^ and Co^2+^ released the oxygen species and formed oxygen vacancies, making the surface of the catalyst ready for the oxygen species adsorption from the gas phase (O_2(gas)_) and accelerating the conversion of the gas phase oxygen species to the adsorbed oxygen species (Reactions (8) and (9)) [7]. The relative content of adsorbed oxygen species of the used CuCo_2_O_4_-N catalysts (40.6%) was significantly decreased compared to the fresh CuCo_2_O_4_-N catalysts (46.8%), and the activity of soot conversion was also decreased. The adsorbed oxygen species played a crucial role in catalytic soot combustion, which could be transformed to active oxygen species such as O_2_, $O_{2}^{-}$ and O^−^ (Reactions (10)–(12)) [52]. Moreover, some lattice oxygen species took over the oxygen vacancies and were transferred into adsorbed oxygen species. The adsorbed oxygen species were spilled over to the soot particle surface and further contributed to the soot combustion reaction [35]. All these active oxygen species could directly participate in catalytic soot combustion reaction via the contact points between catalysts and soot particulates to form CO and CO_2_ [37]. In addition, the released active oxygen species for the conversion of NO oxidation of NO_2_ [42]. Moreover, the Cu^+^ and Co^2+^ species were reoxidized to Cu^2+^ and Co^3+^ due to the replenishment of oxygen vacancies and participation in catalytic soot combustion (Reaction (13)), and a portion of the gaseous NO molecules were also converted to NO_2_ (Reaction (4)) [53]. Hence, the facilitated electron transfer process and abundant adsorbed oxygen species endowed the copper–cobalt spinel catalysts with outstanding soot oxidation activity.

(7) $${C(\mathrm{Soot})+O}^{*}\to \mathrm{CO}x{+O}_{v}$$

(8) $$Cu^{2}{}^{+}+Co^{3+}\to Cu^{+}+Co^{+}+O_{\mathrm{ads}}+O_{v}$$

(9) $$O_{2(gas)}+O_{v}\to O_{ads}$$

(10) $$O_{ads}+e^{-}\to O_{2}^{-}$$

(11) $$O_{2}^{-}+e^{-}\to O^{-}$$

(12) $$O^{-}+e^{-}\to O^{2-}$$

(13) $$Cu^{+}+Co^{2+}+O_{v}\to Cu^{2+}+Co^{3+}+2e^{-}$$

## 5. Conclusions

To obtain a fundamental understanding on the catalytic soot combustion performance of the CuCo_2_O_4_ catalysts prepared with different metal salt precursors, the relationships between the textural and redox properties of the catalysts and soot conversion were investigated.

(1) The tetragonal spinel crystals of CuCo_2_O_4_ were formed for all catalysts, while the single-metal oxide CuO species was formed only on the CuCo_2_O_4_-N and CuCo_2_O_4_-A catalysts. The CuCo_2_O_4_-N catalysts exhibited a higher specific surface area and well-developed pore structure.
(2) The type of metal precursors could also profoundly affect the redox properties on CuCo_2_O_4_ spinel catalysts. The higher relative content of Co^3+^ (38.4%), Cu^2+^ (51.7%) and O_ads_ (46.8%) species were obtained over the CuCo_2_O_4_-N catalysts. Meanwhile, CuCo_2_O_4_-N catalysts (173 °C) showed a lower reduction temperature over CuCo_2_O_4_-A (184 °C) and CuCo_2_O_4_-C (247 °C). The highest amount of surface adsorbed oxygen species (41.2 μmol·g^−1^) was achieved over the CuCo_2_O_4_-N catalysts.
(3) The highest soot conversion activity and CO_2_ selectivity were obtained over the CuCo_2_O_4_-N catalysts regardless of the soot combustion conditions. The effects of the contact mode (loose and tight) and NO addition on soot conversion were also investigated. A good correlation between soot conversion and the textural and redox properties of the catalysts were observed. The reaction mechanisms and pathways of the CuCo_2_O_4_ for catalytic soot combustion were also established.

## Figures and Tables

**Table 1:** Textural properties of the CuCo_2_O_4_-*x* (*x* = N, A and C) catalysts.

Catalysts	S_BET_ (m^2^·g^−1^)	Pore Volume (mm^3^·g^−1^)	Average Pore Diameter (nm)	Average Crystal Size (nm) *
CuCo_2_O_4_-N	2.1	4.5	8.3	38.0
CuCo_2_O_4_-A	2.0	4.2	8.6	38.3
CuCo_2_O_4_-C	1.2	2.5	8.5	38.8

* Calculated by the Scherrer equation, based on the characteristic peak of CuCo_2_O_4_ (3 1 1) crystal face located at the 2θ of 36.8°.

**Table 2:** Redox properties of the CuCo_2_O_4_-*x* (*x* = N, A and C) catalysts.

Catalysts	Co^3+^/Co_total_ (%)	Cu^2+^/Cu_total_ (%)	O_ads_/(O_ads_+ O_latt_) (%)	H_2_ Consumption (mmol·g^−1^)	O_2_ Uptake (μmol·g^−1^)
CuCo_2_O_4_-N	38.4	51.7	46.8	13.4	41.2
CuCo_2_O_4_-A	35.1	48.2	37.2	13.6	27.1
CuCo_2_O_4_-C	33.8	47.8	36.5	14.5	25.6

**Table 3:** Catalytic activity of the CuCo_2_O_4_-*x* (*x* = N, A and C) catalysts for soot combustion under loose contact mode in 10 vol.% O_2_ with balanced N_2_.

Catalysts	10 vol.% O_2_/N_2_			
	T_10_ (°C)	T_50_ (°C)	T_90_ (°C)	S_CO_2__ (%)
CuCo_2_O_4_-N	451	520	558	98.8
CuCo_2_O_4_-A	496	539	569	95.0
CuCo_2_O_4_-C	504	550	594	81.8
No catalysts	530	586	615	58.7

**Table 4:** Catalytic activity of the CuCo_2_O_4_-*x* (*x* = N, A and C) catalysts for soot combustion under the loose contact mode with 1000 ppm NO.

Catalysts	T_10_ (°C)	T_50_ (°C)	T_90_ (°C)	S_CO_2__ (%)
CuCo_2_O_4_-N	349	414	482	96.6
CuCo_2_O_4_-A	381	458	494	94.3
CuCo_2_O_4_-C	392	485	534	79.7
No catalysts	496	575	615	43.6

**Table 5:** Catalytic activity of the CuCo_2_O_4_-N catalysts for soot combustion under the loose and tight contact modes.

Contact Mode	T_10_ (°C)	T_50_ (°C)	T_90_ (°C)	S_CO_2__ (%)
Loose contact	451	520	558	98.8
Loose contact + NO	349	414	482	96.6
Tight contact	385	428	499	100
Tight contact + NO	339	394	448	100

**Table 6:** XPS parameters of CuCo_2_O_4_-N catalyst before and after reaction.

Catalysts	O_ads_/(O_ads_ + O_latt_) (%)
CuCo_2_O_4_-N (fresh)	46.8
CuCo_2_O_4_-N (used)	40.6

## Data Availability

Not applicable.
